# Metabolites and Lipoproteins May Predict the Severity of Early Acute Pancreatitis in a South African Cohort

**DOI:** 10.3390/biomedicines12112431

**Published:** 2024-10-23

**Authors:** Jeanet Mazibuko, Nnenna Elebo, Aurelia A. Williams, Jones Omoshoro-Jones, John W. Devar, Martin Smith, Stefano Cacciatore, Pascaline N. Fru

**Affiliations:** 1 Department of Surgery, School of Clinical Medicine, Faculty of Health Sciences, University of the Witwatersrand, Johannesburg 2193, South Africa; jeanet.mazibuko@wits.ac.za (J.M.); nnenna.elebo@wits.ac.za (N.E.); omsjon@dr.com (J.O.-J.); john.devar@wits.ac.za (J.W.D.); martin.smith@wits.ac.za (M.S.); 2 Bioinformatics Unit, International Centre for Genetic Engineering and Biotechnology, Cape Town 7925, South Africa; stefano.cacciatore@icgeb.org; 3 Human Metabolomics, North-West University, Potchefstroom 2531, South Africa; aurelia.williams@nwu.ac.za; 4 Hepatopancreatobiliary Unit, Department of Surgery, Chris Hani Baragwanath Academic Hospital, Johannesburg 1864, South Africa

**Keywords:** early acute pancreatitis, metabolites, lipoproteins, severity, prediction, nuclear magnetic resonance spectroscopy, South African cohort

## Abstract

**Background:** Acute pancreatitis (AP) can be life-threatening with unpredictable severity. Despite advances in management, its pathogenesis remains unclear. This study investigated metabolites and lipoprotein profiles in AP patients of African descent to understand the underlying pathophysiological conditions so as to inform prognosis and management. **Methods:** Serum samples were collected from 9 healthy controls (HCs) and 30 AP patients (8 with mild AP, 14 with moderately severe AP, and 8 with severe AP) on days 1, 3, 5, and 7 post epigastric pain and subjected to nuclear magnetic resonance (NMR) spectroscopy. Wilcoxon and Kruskal–Wallis rank-sum tests compared numerical covariates. Lipoprotein characterization was performed using the Liposcale test, and Spearman’s rank test assessed data correlations. The *p*-values < 0.05 indicated significance. **Results:** Thirty-eight metabolic signals and information on lipoprotein subclasses were identified from the NMR spectra. The severity of AP correlated with increased levels of 3-hydroxybutyrate and acetoacetate and decreased levels of ascorbate. Distinct metabolic phenotypes were identified and characterized by unique inflammatory and lipoprotein profiles. High-density lipoprotein cholesterol (HDL-C) decreased across all the metabolic phenotypes of AP when compared with the HC, while elevated immediate density lipoprotein cholesterol (IDL-C) and very low-density lipoprotein cholesterol (VLDL-C) levels were observed. Time-dependent changes in metabolites were indicative of responsiveness to therapy. **Conclusions:** Our findings indicate that dysregulated metabolites and lipoproteins can be used to differentiate AP disease state and severity. Furthermore, integrating clinical parameters with data on metabolic and lipoprotein perturbations can contribute to a better understanding of the complex pathophysiology of AP.

## 1. Introduction

Acute pancreatitis (AP) is an inflammatory condition resulting from the autodigestion of the pancreas caused by the sudden release of digestive enzymes before they reach the small intestine. AP clinically presents with nausea, vomiting, abdominal pain, and an increase in amylase and/or lipase in the blood of patients, which is at least three times greater than the normal range [1,2]. The incidence of AP in the Western world is well documented, except in Asia and Latin America [3] and in low- and middle-income countries such as South Africa, which have a paucity of information [4]. Discovery studies from our setting have shown changes in immunological molecules or a lack thereof in various stages of the disease [5,6,7,8], but incidence studies are still lacking. While there is currently no incidence study of AP in South Africa, hospital prevalence studies have shown that there are increasing hospital admissions due to AP [4,9,10]. The most common causes of AP worldwide are biliary-related and alcohol-induced AP [11]. In South Africa, 5% of AP cases are estimated to be due to antiretroviral drugs (ARVs), endoscopic retrograde cholangiopancreatography (ERCP), hypertriglyceridaemia (HTG), or unknown causes [4,10]. Risk factors such as obesity, age, genetic predisposition, and alcohol abuse differ according to the lifestyle of the population [12,13] and play a large role in these changes. AP caused by HTG has surpassed alcohol-induced AP due to dietary habits in China [14], and it will not be surprising if similar trends are reported in African populations as more Western dietary habits are adopted.

AP has diverse presentations with clinical variations and an unpredictable course, which makes diagnosis and hence prognosis difficult due to changes in the threshold of blood enzymes, such as amylase and lipase [15]. The varying degrees of severity make it necessary for patients to be evaluated on time in the emergency area for signs and symptoms, which will aid in the prevention of organ failure and/or death in patients during hospitalization. This is suggestive of a tight therapeutic window, requiring swift responses, which involves monitoring, intervention, and supportive care, which is ideally administered within the first 48–72 h after disease onset [16]. Although several rapid, inexpensive, and reliable tools have been designed to assist clinicians in the triage of AP patients, the sensitivity and specificity challenges together with the threshold change issues associated with blood enzymes means that the prediction of disease severity and progression has not been easy or quick enough [15,17,18,19]. Furthermore, in patients who present diagnostic dilemmas or for whom it is essential to ascertain the extent of necrosis, computed tomography (CT) scans (taken more than 72 h after the onset of acute pancreatitis) may be necessary [20]. The use of computed tomography to diagnose critically ill AP patients might delay treatment, which may result in single or multiple organ failures and death; hence, there is a need for more reliable and fast alternatives.

Although no effective medication exists for the treatment of AP, supportive care, which is dependent on severity, is applied [21]. Unlike mild acute pancreatitis (MAP) patients, moderately severe AP (MSAP) and severe AP (SAP) patients who require specialized care are transferred to the intensive care unit (ICU). Antibiotics are administered to patients with infected necrosis [22]. In biliary-associated pancreatitis, surgical interventions, such as ERCP with early sphincterotomy, can decrease the length of hospital stays and complications [23,24].

The initiation of AP causes alterations in biological processes because the immune system is activated in response to pancreatic injury [5,7]. The activated immune system affects metabolic processes by altering the blood concentration of small molecules, such as sugars, lipids, and amino acids [18,25,26]. The pancreas secretes digestive enzymes and hormones that are crucial for regulating metabolism. Therefore, damage to pancreatic cells due to acute pancreatitis (AP) impacts the metabolome. This suggests that metabolomics of biofluids, such as plasma and serum, may be performed to retrieve information on the “health” status of the pancreas and pancreas-related diseases, aiding in the early detection, diagnosis, and identification of novel biomarkers in pancreatic diseases.

Nuclear magnetic resonance (NMR) spectroscopy is a technique used in metabolomics to quantify a wide range of molecules, including low-molecular-weight metabolites, lipids, and lipoproteins [26,27,28,29]. NMR spectroscopy can detect inflammatory markers, specifically signals from the glycosylation of acute-phase proteins (APPs), like α1-antichymotrypsin, haptoglobin-1, α1-antitrypsin, transferrin, and α1-acid glycoprotein. The carbohydrate modifications on these APPs, including N-acetylglucosamine and N-acetylgalactosamine (GlycA), and N-acetylneuraminic acid (GlycB), produce two distinct NMR signals. Another advantage of NMR spectroscopy over other techniques, such as mass spectrometry, is its high reproducibility, which makes NMR spectroscopy suitable for epidemiologic study [30].

NMR-based metabolomics has been used to distinguish between AP patients and healthy controls; however, metabolomic studies on AP in the African population are lacking. Here, the serum metabolome of AP patients and matched controls was profiled for the first time in an African cohort using NMR spectroscopy. This study aimed to investigate altered metabolites and lipoproteins to better understand the underlying pathophysiological conditions of AP. The study is further supported by the concerns about increasing hospital admissions due to AP [4,9,10], and like many other non-communicable diseases, this burdensome trend is expected to continue [31]. We hypothesize that an understanding of dysregulated metabolites and lipoproteins in AP when considered with clinical parameters could inform a better understanding of AP in our setting and hence lead to improved management of the disease.

## 2. Materials and Methods

### 2.1. Sample Collection and Processing

Ethics clearance for this study was obtained from the Human Research Ethics Committee (Medical) of the University of the Witwatersrand (Ethics No. M190407). We also confirm that all experiments were performed in accordance with the relevant guidelines and regulations. The Revised Atlanta Classification (RAC) of AP of 2012 (MAP, MSAP, or SAP) was used by consulting clinicians to recruit participants at the Chris Hani Baragwanath Academic Hospital (CHBAH). To be included in the study, participants had to be 18 or more years old and self-identified as of African descent. Patients were excluded if they had been diagnosed with autoimmune diseases, were receiving immunotherapy, or were receiving antibiotics within one month of admission. After informed consent, blood samples were collected from participants using red non-anticoagulant BD vacutainer^®^ blood collection tubes (BD Biosciences, Franklin Lakes, NJ, USA). Four milliliters (4 mL) of blood were drawn from eligible patients as previously described by Nalisa and colleagues on days 1, 3, 5, and 7 post epigastric pain, enabling time points and longitudinal analysis of the metabolic changes [7]. Patients typically reported to the hospital on day 3 of pain.

The clinical data were obtained from the patients’ hospital files via the National Health Laboratory Services (NHLS) Trakcare Web Results, version L6.10, which is captured in the RedCap^®^ (Nashville, TN, USA) database. Captured data included demographics, body mass index (BMI), AP aetiology, AP severity, clinical test results, and comorbidities. Healthy volunteers were recruited as controls and were age- and sex-matched with the AP patients.

### 2.2. Serum Sample Preparation

The collected blood samples were allowed to stand upright for 30 min at room temperature in blood collection tubes and then centrifuged at 1734× *g* at 4 °C for 30 min. The serum was aliquoted into microfuge tubes (500 µL) and stored at −80 °C until analysis.

The frozen serum samples stored at −80 °C, were thawed at room temperature. A working solution was prepared by adding 300 µL of thawed serum to 300 µL of a solution consisting of 0.75 M potassium phosphate buffer (pH 7.4), 5.81 mM trimethylsilyl-2,2,3,3-tetradeuteropropionic acid (TSP; Sigma–Aldrich, St. Louis, MO, USA), and a trace amount of sodium azide (65 mg dissolved in deuterium oxide) to prevent bacterial growth. The samples were vortexed to obtain a homogenous mixture. A final volume of 540 µL of each sample was transferred to a 5 mm NMR tube (Wilmad Lab Glass, Vineland, NJ, USA) for analysis. Sample preparation and analysis were performed at the Centre for Human Metabolomics, Potchefstroom Campus, North-West University, South Africa.

### 2.3. NMR Analysis

The NMR tubes containing the respective samples were loaded on a 500 MHz Bruker Avance III HD NMR spectrometer equipped with a triple resonance inverse 1H probe head and x, y, z gradient coils. Afterwards, one-dimensional (1D) proton (1H)-NMR spectra were acquired. A standard nuclear Overhauser effect spectroscopy (NOESY) pulse sequence with presat (noesygppr1d.comp) was used on both the metabolite and lipid extract samples. The NOESY was used to detect the signals of both small metabolites and high-molecular-weight macromolecules, such as lipoproteins. Additionally, a standard diffusion-edited (DIFF) pulse sequence (ledbpgppr2s1d) was used to detect only high-molecular-weight macromolecules, such as lipoproteins. Pooled AP samples of different severities were used as a quality control sample and were included in each batch for qualitative assessment of repeatability by overlaying the raw spectra.

### 2.4. NMR Profiling

NMR spectroscopy was used to quantify signals from the samples, which were subsequently identified and quantified. The peaks of the identified metabolites were fitted by combining a local baseline and Voigt functions based on the multiplicity of the NMR signal. The assignment of the quantified signals is reported in Table S1. To validate the efficacy of the different deconvolution models, the root-mean-square deviation was determined. The absolute concentration of each metabolite was calculated according to a previously reported equation [32]. The number of protons contributing to the unknown signals was imputed to 1. The concentration of carbohydrates was also estimated by considering the equilibrium between their cyclic forms.

GlycA and GlycB signals were quantified by integrating the areas between 2.00 and 2.05 ppm and between 2.09 and 2.05 ppm, respectively. GlycA is measured as an NMR signal of post-translational modification of glycosylated acute-phase proteins released during inflammation [33]. Given our previous experience [26] on lipid dysregulation in pancreatic cancer (PC) and the fact that AP predisposes patients to PC [34], we went on to quantify the effect of AP on the lipid profile within this cohort. This was performed using the Liposcale test (Biosfer TesLab, Reus, Spain), to determine lipoprotein parameters, high-density lipoprotein (HDL), low-density lipoprotein (LDL), and very low-density lipoprotein (VLDL) particle number, size, and lipid concentration of each subtype [35]. The assay was performed as previously described [26]. Each of the DIFF spectra in the range between 0.1 and 9.5 ppm, excluding the regions corresponding to the water signals between 4.40 and 5.00 ppm, was segmented into 0.001 ppm chemical shift bins, and the corresponding spectral areas under the curve yielded a total of 8800 variables.

### 2.5. Statistics and Data Analysis

Statistical analysis and graphical illustrations of the data were generated in R (version 4.3.0) and R studio (version 2023.12.1+402) software using scripts developed in-house. Wilcoxon and Kruskal–Wallis rank-sum tests were used to compare differences in numerical covariates (e.g., age and metabolite concentration). Fisher’s exact test was used to assess differences between categorical variables (e.g., gender), and Spearman’s rank test was then used to calculate the correlation coefficient (rho) between variables. The *p*-values < 0.05 were considered significant, and to account for multiple testing, a false discovery rate (FDR) of <10% was applied.

The KODAMA algorithm, which allows for unsupervised extraction of features and enables analysis of noisy datasets of high dimension, was used to facilitate the identification of patterns representing underlying metabolic phenotypes in all samples [36,37,38]. The partition around medoids (PAMs) clustering [39] was applied to the KODAMA scores using the silhouette algorithm 10 [40] to verify the results obtained. The silhouette median value was utilized to assess the ideal number of clusters, ranging from 2 to 10.

Partial least squares (PLSs) analysis was then performed to predict the free cholesterol and cholesterol ester ratio from the DIFF spectra as described by Elebo and colleagues [26]. Briefly, each DIFF spectrum was segmented into 0.001 ppm chemical shift bins in the range between 0.1 and 9.5 ppm, excluding the regions corresponding to the water signals between 4.40 and 5.00 ppm. A training set of the PC samples DIFF spectra retrieved from the study by Elebo and colleagues [26] and associated information on the free cholesterol and cholesterol ester ratio was used to build a PLS model using the function pls.kodama in the KODAMA R package. The PLS model was then applied to the DIFF spectra of samples from patients with AP.

## 3. Results

### 3.1. Demographics and Clinical Characteristics

Thirty patients with AP (eight with MAP, fourteen with MSAP, and eight with SAP) and nine healthy controls (HCs) were enrolled in the study. Patients were conveniently sampled based on study criteria, referencing similar sample sizes from previous studies that yielded clinically and statistically relevant data [6,41,42]. There were no statistically significant differences in BMI, age, and gender across the four groups, as shown in Table 1. The primary aetiology of AP in this study was alcohol-induced AP (16/30 = 53%), which occurred mostly in males (12/16 = 75%). Biliary-associated AP was the second most common aetiology (12/30 = 40%), with the majority being female (9/12 = 75%). ARV-induced AP occurred in 7% of the population (2/30). Demographic and clinical features are summarized according to the aetiology in Table S2.

The incidence of organ dysfunction (OD) increased with the severity of AP, with 50% (n = 3/6) of MSAP patients having respiratory OD, while 67% (4/6) was found in the SAP group. Imaging findings consistent with local complications also denoted as complications, such as pancreatic collection and wall-off necrosis, were found in 33% (4/12) of patients with severe AP (i.e., MSAP and SAP). As expected, admissions to the intensive care unit (ICU) and hospital deaths were prevalent among SAP patients.

**Figure 1:**
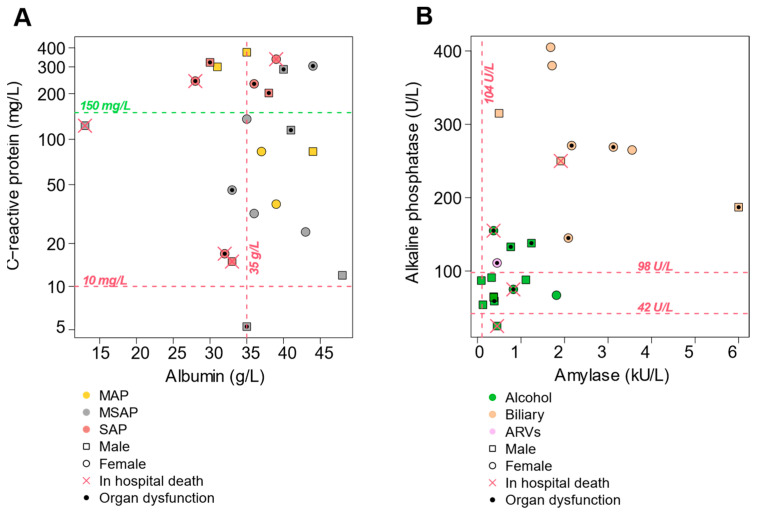
Plot showing (**A**) the correlation between the serum ALB concentration and the serum CRP concentration. The top and middle right quadrants are patients with a Glasgow Prognostic Score (GPS) = 1 with a CRP concentration > 150 mg/L and an ALB concentration > 35 g/L, while the left quadrants are patients with a GPS = 2. Although the GPS in the top right quadrant is 1, patients are characterized by elevated organ dysfunction. (**B**) The correlation between amylase and ALP. Decreased levels of both amylases and alkaline phosphatase (ALP) are associated with alcohol-associated AP, while increased levels are linked to biliary-associated AP. A GPS of 0, 1, or 2 indicates neither, one, or both abnormalities. ALB: albumin, CRP: C-reactive protein, GPS: Glasgow Prognostic Score.

No statistically significant differences were found between the severity of AP and routine biochemical tests, including amylase, lipase, CRP, hemoglobin, hematocrit, and platelets (Table S3). The Glasgow Prognostic Score (GPS) was calculated to evaluate the systemic inflammatory response using the serum concentrations of CRP and albumin [43]: 32% (7/22) of AP patients had a GPS of 2 (severe inflammation), 64% (14/22) of AP patients had a GPS of 1 (moderate inflammation), and only one patient had a GPS of 0 (mild inflammation) (Figure 1A). One-third (10/30) of the patients in our cohort were affected by hypoalbuminemia, and almost all AP patients had CRP levels above 10 mg/L. AP patients with a CRP concentration higher than 150 mg/L and an albumin concentration lower than 35 g/L were more likely to experience in-hospital death or organ dysfunction (11/13 = 85%). All patients with SAP had a GPS of 2 or CRP higher than 150 mg/L.

In this cohort, amylase and alkaline phosphatase (ALP) were significantly (*p* < 0.001) elevated in biliary-associated AP. Their combined levels could distinguish biliary-associated AP from alcohol-associated AP, as shown in Figure 1B. The median levels of liver function markers, such as total bilirubin, conjugated bilirubin, alanine transaminase (ALT), aspartate transaminase (AST), and ALP, were elevated beyond the normal physiological range in patients with SAP. A comparison of biochemical tests revealed significant differences in both amylase (*p*-value < 0.001, FDR = 0.008) and ALP (*p*-value < 0.001, FDR = 0.003) across the three different aetiologies of acute pancreatitis (Table S4). Furthermore, there were no statistically significant differences observed for a comparative analysis between the different AP severity groups and biochemical tests performed in biliary-associated AP (Table S5) and alcohol-associated AP (Table S6).

### 3.2. Metabolites and Acute Pancreatitis Severity

NMR spectroscopy was used to profile all the concentrations of metabolites and lipids, and 38 metabolic signals, including lipid classes and the NMR inflammatory markers GlycA and GlycB, were detected. To delineate the metabolic signatures of AP, the Spearman correlation test was performed to link metabolic parameters to the different groups in the following rank order: HC = 0, MAP = 1, MSAP = 2, and SAP = 3 (Table 2). Elevated levels of 3-hydroxybutyrate (3-HB), acetoacetate, phenylalanine, mannose, and lactate, as well as decreased concentrations of ascorbate, methanol, ethanol, and glutamine, were observed with increasing AP severity. Additionally, AP severity showed a large alteration in the lipid profile with increasing lipid alpha-CH_2_ and decreasing cholesterol, lipid =CH-CH_2_-CH=, lipid beta-CH_2,_ and lipid CH_3_ with disease severity, as well as a decrease in the serum levels of protein (protein NH). Although not statistically significant, the inflammatory markers GlycA and GlycB showed a positive correlation indicating the higher inflammatory processes in AP patients compared to HC. Indeed, the comparison analysis between AP and HC showed a higher statistically significant value in AP for GlycA (*p*-value = 0.00205) and GlycB (*p*-value = 0.00183). GlycA and GlycB have previously been associated with the value of CRP [25]. In AP patients, a statistically significant positive correlation was observed only for CRP and GlycB (*p*-value = 0.043). The correlation between CRP and GlycA was not statistically significant. The aetiology of AP, i.e., alcohol or biliary-induced AP, had only a mild impact on the metabolic profile (Table S7).

### 3.3. The Relationship between Metabolic Phenotypes and Clinical Outcomes in Acute Pancreatitis Patients

**Figure 2:**
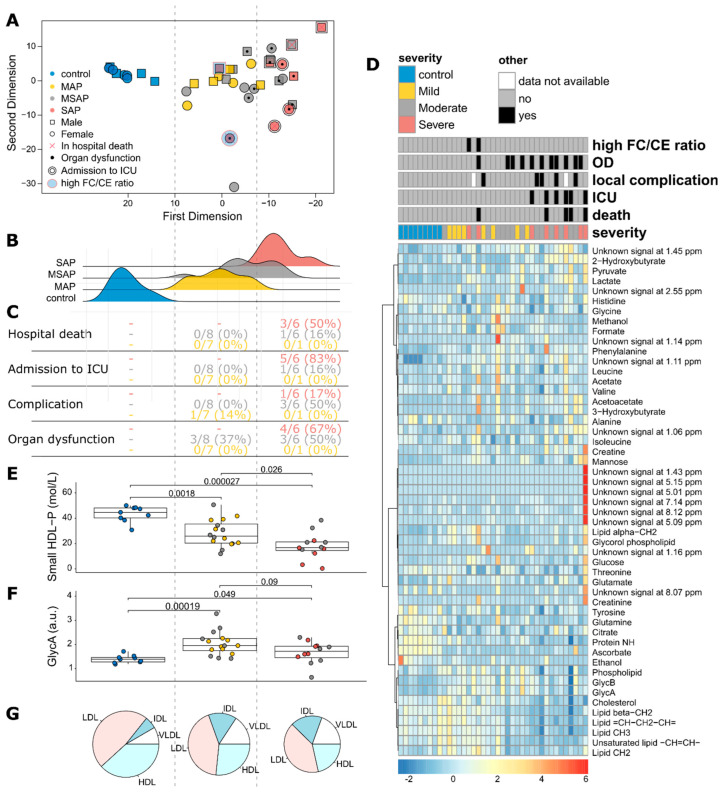
Overview of the metabolic phenotypes of AP and their effects. (**A**) Unsupervised analysis of metabolites from healthy controls (HCs, n = 9), mild AP (MAP, n = 8), moderately severe AP (MSAP, n = 14), and severe AP (SAP, n = 8) patients revealed two distinct clusters. MAP and most MSAP patients formed one cluster, while SAP patients, except for two with elevated FC/CE ratios and 5 MSAP patients, formed the other. (**B**) The distribution of AP and HC groups shows clear separation, with HCs and SAP patients on opposite sides. MAP and MSAP patients were centrally located, with some overlap. (**C**) Clinical feature comparison showed MAP patients (yellow) experienced the fewest complications, while SAP patients (red) faced the most severe outcomes, including death. MSAP patients had a higher overall complication rate (50%) compared to SAP patients (17%), but less organ dysfunction (43% vs. 67%). (**D**) A heatmap revealed distinct lipid profile differences across AP groups, indicated by color changes from blue to red. (**E**,**F**) Small HDL-P levels decreased with increasing AP severity, while GlycA levels increased in mild AP and moderately decreased in severe AP. (**G**) Cholesterol-related lipid distribution showed decreased HDL-C and increased VLDL-C and IDL-C from HCs to SAP patients, suggesting impaired anti-inflammatory function, reduced lipoprotein synthesis, and compromised immune functions in AP. HDL: High-density lipoprotein, HDL-P: HDL-Particle, LDL: Low-density lipoprotein, VLDL: Very-Low Density Lipoprotein, IDL: immediate-density lipoprotein, FC/CE: free cholesterol/cholesterol ester ratio.

Unsupervised KODAMA analysis was performed to visualize patterns between metabolic phenotypes (metabotype) and disease severity, as shown in Figure 2A. This operation separated the patient cohort into three different metabotypes. A distinct metabolic profile was observed for the healthy controls compared to the AP patients. Notably, a clear separation was also evident between patients with MAP and those with SAP. Interestingly, two patients with SAP showed a metabolic profile similar to that of patients with MAP. Further analysis indicated that these patients had elevated free cholesterol/cholesterol ester ratios, which could be due to cholestasis linked to lipoprotein X (LpX). Cholestasis is characterized by the presence of Lpx [44], which can mask the SAP phenotype. The metabolic profiles of AP patients with intermediate severity (i.e., MSAP) overlapped in both the MAP and SAP patient profiles (Figure 2B). However, MSAP patients with a phenotype closer to that of SAP patients had more adverse events than those closer to the MAP group (Figure 2C). However, the SAP patients, as expected, had the most adverse events. Some of the adverse events were complications, such as infection and pseudocysts, organ dysfunction (OD), and admission to the ICU. Notably, increases in lactate, shown by the color changes from blue through yellow in the heatmap (Figure 2D), which are prevalent in SAP patients, also correspond to increased OD, local complications, ICU admission, and death. A comparison of the metabolites (Table S8) and lipoproteins (Table S9) with inverted first-dimension KODAMA showed variations in metabolite concentrations, such as mannose, ascorbate, phenylalanine, and lipoprotein features.

A further comparison of the clinical features and the metabolite profile showed that the lipid profile distinctively separated the metabolic phenotypes of AP patients based on their severity, with an overall decrease in lipid levels with increasing AP severity (Figure 2D). The concentration of high-density lipoprotein particles (HDL-Ps) appeared to constitute the difference in the dyslipidaemia found between the metabolic phenotypes, with a notable decrease with increasing AP severity (Figure 2E). The inflammatory marker GlycA increased across the groups and was highest in the MSAP patients (Figure 2F). After further characterization of cholesterol-related lipids, dysregulation of HDL (decreasing with severity) and IDL and VLDL (both increasing with severity) were shown to be linked to AP severity (Figure 2G).

### 3.4. Trend Analysis

**Figure 3:**
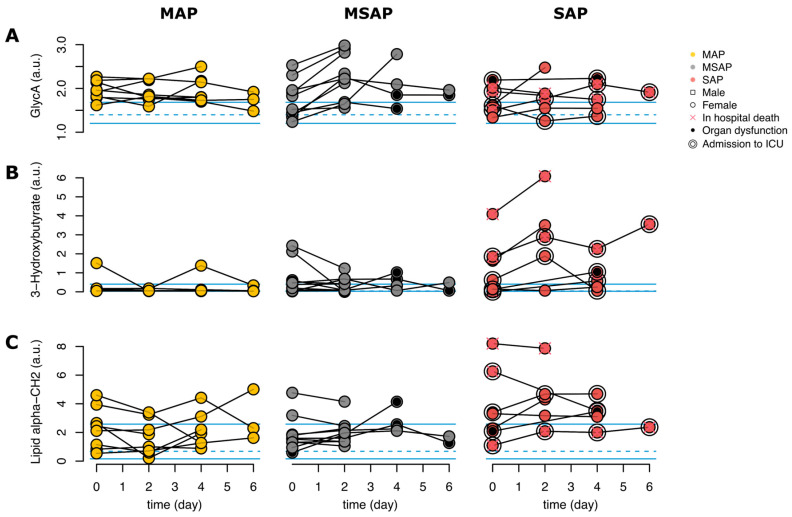
Trends in selected metabolic parameters. (**A**) The pattern for GlycA in the severity groups of AP over all time points. (**B**) Assessment of 3-hydroxybutyrate levels in the severity groups of AP over all time points. (**C**) Describes the pattern for lipid alpha-CH_2_ in the severity groups of AP over all time points.

To further understand the association of metabolites and lipoproteins with AP severity, a trend analysis (Figure 3) was performed to analyze three representative molecules (GlycA, 3-HB, and lipid alpha-CH_2_). The daily changes were calculated as the difference in concentration between two time points divided by the number of days in between. The daily changes in AP severity (Table S10), worse clinical outcomes (i.e., in-hospital death or admission to the ICU) (Table S11), and organ dysfunction (Table S12) were analyzed. The comparison analysis of the daily changes in AP severity revealed an increase in GlycA levels during the hospitalization in the MSAP group (*p*-value = 0.004), as illustrated in Figure 3A. In this pilot study, the daily change in the ketoacidosis metabolite 3-HB (*p*-value = 0.003) was shown to be significantly linked to worse clinical outcomes in AP patients, as shown in Figure 3B, and within the severe group, this metabolite seemed to increase with time from day 1 to day 7. Elevated levels of 3-HB were observed in SAP patients compared to patients in the other severity groups. Lipid alpha-CH_2_ was dysregulated in AP patients with organ dysfunction (Figure 3C). Elevated levels of alpha-CH_2_ (*p*-value = 0.007) were observed in SAP patients compared to MSAP patients and MAP patients. Within patients, it appeared to be consistent over time.

## 4. Discussion

In this preliminary study, the demographics, clinicopathological characteristics, and metabolite and lipoprotein profiles of 30 AP patients were analyzed to assess the relationships between these parameters and AP severity and clinical outcome. Importantly, the identification of a metabotype that could help in the prognosis of AP disease severity was envisaged.

Approximately 57% (17/30) of the AP patients were obese, with a BMI of 32 kg/m^2^ and a median IQR of 30.9–39.8 kg/m^2^ in SAP. Notably, obesity was more common in patients with biliary-associated AP. An increase in BMI of approximately 83% (10/12) was observed for biliary-associated AP patients. Previous studies have shown that comorbidities, such as obesity, affect the clinical outcomes of AP [45], and this is also true for the cohort evaluated.

Similar to other studies, the findings from this study revealed that alcohol-induced acute pancreatitis is the leading cause of AP in males, whereas biliary-associated AP is more prevalent in females [46,47]. In contrast, a retrospective study in a Western population showed that biliary-induced AP, which is prevalent in females, is the main cause of AP, followed by alcohol, which is predominant in males [13]. Furthermore, more than 50% of the female AP patients in this study were living with HIV/AIDS and receiving antiretroviral therapy (ART). The incidence of acute pancreatitis is extremely high in people living with HIV/AIDS (PLWHA), which could be due to the long-term use of antiretroviral therapy (ART) medications, such as corticosteroids and sulphonamides [48]. Importantly, the aetiology of AP did not have a notable impact on the metabolic profile, possibly because the course of the disease is not usually affected by the aetiology [49].

Serum amylase and lipase are routine tests used for the diagnosis of AP [50]. According to the South African NHLS report, the physiological concentrations of amylase and lipase range from 28 to 110 U/L and 13 to 60 U/L, respectively [51]. Our study participants had higher concentrations of both amylase and lipase, approximately three times above the normal limit, but no statistically significant differences were found between the groups. Elevated levels of amylases and ALP were observed in our study population, which suggests liver damage or a blocked bile duct in biliary pancreatitis [52]; hence, these findings could be vital for distinguishing AP from alcohol-induced AP [53]. Indeed, this study confirmed that the combination of amylase and ALP can be used to differentiate biliary-associated AP from alcohol-associated AP [54].

This study further confirmed the previously reported direct association of hypoalbuminemia with severity and mortality in AP patients [55]. Most SAP patients also generally had extremely high CRP levels. The results revealed that MSAP patients had the most complications and organ failures from pancreatic necrosis (21%, 3/14), compared to 16% (1/6) for SAP patients, which ultimately resulted in organ failure if unresolved. However, SAP patients had the greatest number of deaths, which is not surprising given that SAP is characterized by persistent organ failure for more than 48 h, unlike in the MSAP. The disease progression leading to organ failure and/or death in AP is usually due to SIRS, which is a response to an infection or an injury in the pancreas.

In this study, the physiological metabotype was able to distinguish between the mild and severe groups of patients. AP patients undergo hypermetabolic conditions, which dysregulate amino acid, lipid, and glucose metabolisms [56]. The concentrations of metabolites, such as lactate, phenylalanine, mannose, acetoacetate, and 3-HB, increased, while the concentrations of ascorbate, methanol, ethanol, and glutamine decreased with increasing AP severity in this cohort.

Lactate levels are most positively correlated with AP severity. Oxygen deficiency and increased energy consumption enhance anaerobic glycolysis, which occurs in the early phase of AP, ultimately leading to elevated levels of lactate, a glycolytic metabolite [57]. An increased lactate concentration has been associated with persistent organ failure in AP patients [58] and is independently associated with poor outcomes and death [59]. Although lactate is a useful marker in disease mortality and severity, it is an unreliable marker for tissue hypoxia/hypoperfusion in critically ill patients [60], suggesting that the link to AP may be non-specific. Increases in mannose levels are also positively correlated with AP severity, which is not surprising given the involvement of mannose in the glycolytic pathway [61].

Organ failure and mortality have been associated with increased phenylalanine levels in the plasma of AP patients [62,63]. Increases in phenylalanine levels could be a result of the scavenging of tetrahydrobiopterin, a key co-factor involved in the conversion of phenylalanine to tyrosine [64]. Dysregulated amino acid metabolism leads to the release of excess acetyl-CoA into the tricarboxylic acid (TCA) cycle, which is converted to ketone bodies, such as 3-HB [57]. Ketones, which are products of the oxidative pathway of alcohol metabolites, could be predictive of alcohol-induced AP [41,65]. Elevated levels of 3-HB in AP have been shown to differentiate between MAP and SAP [61] and are associated with poor survival rates in patients with pancreatic cancer [26]. In this study, 3-HB increased with severity and was found to persist in SAP patients over time when a trend analysis was performed. The enzyme hydroxybutyrate dehydrogenase, which catalyzes the breakdown of 3-HB to acetoacetate, has been shown to significantly increase with organ failure and SIRS [66]. The increasing levels of both 3-HB and acetoacetate with increasing severity are suggestive of the reversible reaction between the two molecules with the possibility of equilibrium. Hence, both 3-HB and acetoacetate could be used as clinical biomarkers to predict worse outcomes.

It is not surprising that ascorbate (vitamin C) levels decreased in this study with increasing AP severity, a phenomenon that has been previously reported [67]. High doses of ascorbate have shown therapeutic efficacy in AP [68]. Glutamine levels also significantly decreased with increasing AP severity. The consumption of glutamine by immune cells is similar, if not more than that of glucose. It is essential for processes such as the proliferation of immune cells and the production of cytokines, among others [69]. Inflammatory and immunocompromised patients, therefore, inevitably experience decreasing levels with severity, as was the case here.

Similar to the other metabolites, the levels of two alcohols, methanol and ethanol, decreased with increasing AP severity but were not significantly different between biliary- and alcohol-induced AP patients (Table 2), as would have been expected. This lack of a strong metabolic signature is supported by the fact that irrespective of the cause of AP, the trajectory is usually the same [49]; hence, the aetiology does not influence the metabotype. In addition, the mechanisms underlying the aetiology of AP have not been extensively elucidated, especially for alcohol-induced AP, and could shed light on this. This study distinctly revealed a dyslipidaemic profile in AP patients based on severity. Serum lipid levels are associated with the severity of acute pancreatitis [70]. The small HDL-P concentration decreased with increasing severity of AP. Studies have shown that small HDL-P plays a vital role in ATP-binding Cassette transporter A 1 (ABCA-1)-mediated cholesterol efflux from macrophages, which leads to the deployment of intracellular cholesterol to the plasma membrane [71]. HDL-C decreased from HCs to SAP patients, while IDL and VLDL increased with severity. Recent studies have demonstrated that decreased HDL-C levels are an independent prognostic factor for adverse outcomes, such as persistent organ failure and death [70]. HDL-C possesses anti-inflammatory properties; hence, decreased amounts of HDL-C could be latent for AP severity [72]. Oxidized VLDL is a marker of oxidative stress, which inhibits lipoprotein lipase, and is responsible for triglyceride (TG) metabolism [73]. Notably, serum triglyceride levels have also been associated with severity in acute biliary pancreatitis [74]. Elevated VLDL levels are associated with HTG, which is the third leading cause of AP and correlates with increased severity [14], raising the question of whether there could be HTG-associated AP in the study setting. The protein concentration decreased with increasing severity in this study, confirming that AP promotes protein catabolism, which involves the breakdown of proteins into amino acids [75]. Hence, the presence of pancreatic inflammation could lead to an alteration in the level of metabolites [61].

This study confirmed that inflammation plays a vital role in determining AP severity [76]. AP patients with CRP > 150 mg/L and albumin < 35 g/L were more likely to die in-hospital or have organ dysfunction. Furthermore, GlycA was most elevated in MSAP patients, with a slight increase in SAP patients, suggesting increased inflammation in MSAP patients compared to MAP and SAP patients. Although the differences in GlycA between the groups in this study were not significant, it is notable that in a recent study, GlycA could be used as a novel diagnostic marker of inflammation in AP patients compared to healthy controls [77]. Although there is a notable similarity between GlycA (and GlycB) and CRP as markers of inflammation, the correlation analysis between GlycA/GlycB and CRP showed that they are not strongly associated. GlycA/GlycB and CRP capture different aspects of the inflammatory response. CRP is an “early” APP, and the proteins that contribute the most to the GlycA and GlycB signal rise later in the acute phase response. Another major difference between the CRP and GlycA is that CRP is a single APP whereas GlycA reflects the integrated concentrations and glycosylation states of multiple acute-phase proteins [78].

The transcription factor NF-kB has been shown to promote the expression of genes involved in inflammation, which are vital in various AP stages [79]. AP is an immune disorder characterized by the release of either anti-inflammatory or proinflammatory cytokines [70]. The impaired anti-inflammatory function might be linked to reduced HDL-C levels in AP, leading to increased free fatty acids (FFAs), which produce an acidic microenvironment [70]. HDL-C also possesses antioxidant ability, which is mediated by paraoxonase-1 activity in AP [80]. Notably, the presence of FFAs results from the breakdown of TG, including chylomicrons, and is associated with HTG AP. TGs are inherently not toxic, but the resultant lipotoxicity from FFAs causes the release of intracellular calcium, resulting in acinar cell injury [14].

The use of RAC for classifying patients in this study and other classifications, such as the determinant-based classification (DBC), assist in stratifying patients for improved care and not to predict severity [81]. The RAC is known to better determine the length of a hospital stay, while the DBC more accurately predicts the need for intervention. However, using both or multiple classifications is not ideal in clinical settings. Additionally, these classifications need to be improved to account for the dynamic nature of organ dysfunction in AP, consider its complex pathophysiology, and predict potential transitions between classes [81,82]. As a result, it is not surprising that the main classifications in this study were between MAP and SAP, with MSAP patients being transitory between the two. Additionally, conditions like cholestasis, which may have initially triggered AP, resulted in two SAP patients being classified as having MAP. One MAP patient was on the borderline between MAP and SAP, underscoring the dynamic and complex pathophysiology of AP.

Limitations of this work include the fact that “sick” control samples of patients who presented with other inflammatory diseases that could result in the production of similar metabolites or lipoprotein profiles were not included in this study; hence, the specificity of the metabolic changes could not be fully ascertained. An example is the elevated lactate levels, which are commonly associated with severe diseases in general and may not be specific for AP alone [60]. However, given that metabolites in diseases could vary due to differences in the disease course, metabolomics testing stands to differentiate these markers due to the differing specificities and the associated time-bound nature [18]. This pilot study involved 30 patients with AP and demonstrated statistically significant differences that correlated with disease severity, consistent with findings from other metabolomic studies, including those focused on AP, which have provided conclusive results [41,42,83]. However, increasing the sample size and conducting an independent validation are necessary to draw more robust conclusions and improve the comparisons between the groups. As previously mentioned, NMR-based metabolomics has been used to differentiate between various AP groups; however, metabolomic studies on AP in African populations are still lacking. To thoroughly examine metabolomic changes in samples from African individuals, an exploratory untargeted NMR study was warranted. The NMR results provided valuable insights into the classes of molecules that differed significantly between AP severity groups and can now serve as a foundation for targeted mass spectrometry-based metabolomics experiments, where low abundant molecules can be detected with greater accuracy. Incorporating these and other ethnic groups to account for genetic, dietary, and environmental differences in metabolomic changes, along with studies on protein expression and modifications, will provide a more comprehensive understanding of the underlying biological mechanisms. These aspects will be explored in future research.

## 5. Conclusions

Taken together, the dysregulated metabolites not only differentiated disease state and severity but also reflected the immune deterioration associated with AP. The findings, although not unique to our cohort, are the first to concurrently analyze the lipoprotein status of patients and to represent findings from an African population. Despite the dynamic and inherent complexity of AP and the ongoing challenge of identifying its severe form, these preliminary findings suggest that metabolites and lipoproteins may be useful in determining AP severity in this cohort. When combined with existing classification systems, these findings deepen our understanding of the pathophysiology of AP and have the potential to improve care early in severe AP.

## Figures and Tables

**Table 1:** Demographic and clinical characteristics of acute pancreatitis patients.

Feature	Control (n = 9)	MAP (n = 8)	MSAP (n = 14)	SAP (n = 8)	*p*-Value
BMI (kg/m^2^), median [IQR]	30.4 [23.6 32.8]	24.8 [24.7 44.3]	27 [23.2 30.5]	32 [30.9 39.8]	0.515
Age (years), median [IQR]	43 [30 54]	43.5 [36.5 54.8]	38 [32 43.5]	48 [32.2 57.2]	0.613
Gender					0.100
F, n (%)	5 (55.6)	4 (50.0)	7 (50.0)	4 (50.0)	
M, n (%)	4 (44.4)	4 (50.0)	7 (50.0)	4 (50.0)	
Aetiology of pancreatitis					
Biliary, n (%)		4 (50.0)	5 (35.7)	3 (37.5)	
Alcohol, n (%)		4 (50.0)	8 (57.1)	4 (50.0)	
ARVs, n (%)		0 (0.0)	1 (7.1)	1 (12.5)	
Days of hospitalization, median [IQR]		10.5 [4.8 15]	9 [4.8 11]	8 [8 10]	0.864
no, n (%)		7 (87.5)	10 (71.4)	8 (100.0)	
yes, n (%)		1 (12.5)	4 (28.6)	0 (0.0)	
Diabetic					0.100
no, n (%)		8 (100.0)	13 (92.9)	8 (100.0)	
yes, n (%)		0 (0.0)	1 (7.1)	0 (0.0)	
HIV					0.650
no, n (%)		7 (87.5)	10 (71.4)	7 (87.5)	
yes, n (%)		1 (12.5)	4 (28.6)	1 (12.5)	
Organ dysfunction					0.027
no, n (%)		8 (100.0)	8 (57.1)	3 (37.5)	
renal, n (%)		0 (0.0)	0 (0.0)	1 (12.5)	
respiratory, n (%)		0 (0.0)	3 (21.4)	4 (50.0)	
transient renal, n (%)		0 (0.0)	3 (21.4)	0 (0.0)	
Local Complications					0.878
ANP, n (%)		1 (12.5)	0 (0.0)	0 (0.0)	
no, n (%)		7 (87.5)	10 (76.9)	6 (85.7)	
pancreatic collection, n (%)		0 (0.0)	1 (7.7)	0 (0.0)	
peripancreatic fatty stranding, n (%)		0 (0.0)	0 (0.0)	1 (14.3)	
wall off necrosis; ANC, n (%)		0 (0.0)	1 (7.7)	0 (0.0)	
yes, but not reported, n (%)		0 (0.0)	1 (7.7)	0 (0.0)	
Admission to ICU					0.004
no, n (%)		8 (100.0)	13 (92.9)	3 (37.5)	
yes, n (%)		0 (0.0)	1 (7.1)	5 (62.5)	
Surgical procedures					0.566
cholecystectomy, n (%)		2 (28.6)	1 (7.7)	0 (0.0)	
ERCP, n (%)		0 (0.0)	1 (7.7)	1 (14.3)	
None, n (%)		5 (71.4)	11 (84.6)	6 (85.7)	
Hospital death					0.02
no, n (%)		8 (100.0)	13 (92.9)	4 (50.0)	
yes, n (%)		0 (0.0)	1 (7.1)	4 (50.0)	

Abbreviations: ARVs, antiretrovirals; HIV, human immunodeficiency virus; ANP, atrial natriuretic peptide; ANC, absolute neutrophil count; ICU, intensive care unit; ERCP, endoscopic retrograde cholangiopancreatography; IQR, interquartile range.

**Table 2:** Correlations of metabolite concentrations with acute pancreatitis severity. The data were analyzed using the Spearman correlation test.

Feature	Rho	*p*-Value	FDR
Formate	0.04	0.793	0.809
Unknown signal at 8.12 ppm	0.01	0.951	0.951
Unknown signal at 8.07 ppm	−0.08	0.630	0.684
Phenylalanine	0.54	<0.001	0.028
Tyrosine	−0.13	0.443	0.525
Unknown signal at 7.14 ppm	0.19	0.237	0.318
Histidine	−0.1	0.557	0.631
Glucose	0.24	0.140	0.238
Mannose	0.57	<0.001	0.015
Unknown signal at 5.15 ppm	0.23	0.155	0.239
Unknown signal at 5.09 ppm	0.16	0.330	0.421
Unknown signal at 5.01 ppm	0.23	0.155	0.239
Ascorbate	−0.46	0.003	0.013
Threonine	−0.33	0.043	0.104
Lactate	0.67	<0.001	<0.001
Creatinine	0.2	0.218	0.309
Creatine	0.3	0.064	0.143
Glycine	0.06	0.717	0.746
Methanol	−0.46	0.001	0.013
Unknown signal at 2.55 ppm	0.25	0.130	0.229
Citrate	−0.16	0.330	0.421
Glutamine	−0.55	<0.001	0.003
Pyruvate	0.2	0.230	0.317
Glutamate	0.25	0.126	0.229
Acetoacetate	0.63	<0.001	<0.001
Acetate	0.28	0.089	0.169
Alanine	−0.29	0.077	0.158
Unknown signal at 1.45 ppm	0.44	0.005	0.016
Unknown signal at 1.43 ppm	0.23	0.155	0.239
3-Hydroxybutyrate	0.46	<0.001	0.013
Ethanol	−0.64	<0.001	<0.001
Unknown signal at 1.16 ppm	−0.07	0.686	0.729
Unknown signal at 1.14 ppm	0.31	0.054	0.124
Unknown signal at 1.11 ppm	0.47	0.003	0.013
Unknown signal at 1.06 ppm	0.4	0.011	0.032
Valine	−0.14	0.411	0.511
Isoleucine	0.22	0.018	0.276
Leucine	0.11	0.049	0.564
2-Hydroxybutyrate	0.34	0.032	0.081
Protein NH	−0.75	<0.001	<0.001
Unsaturated lipid -CH=CH-	−0.35	0.029	0.077
Lipid alpha-CH_2_	0.45	0.004	0.013
Cholesterol	−0.43	0.006	0.017
Lipid =CH-CH_2_-CH=	−0.55	<0.001	0.002
Glycorol phospholipid	0.21	0.199	0.289
Phospholipid	−0.13	0.440	0.525
Lipid beta-CH_2_	−0.52	<0.001	0.004
Lipid CH_2_	0.09	0.599	0.664
Lipid CH_3_	−0.44	0.005	0.016
GlycB	0.28	0.085	0.167
GlycA	0.29	0.074	0.158

Abbreviations: FDR, false discovery rate.

## Data Availability

All data produced in the present study are available upon reasonable request to the authors. NMR spectra were deposited in the Metabolight database (https://www.ebi.ac.uk/metabolights/MTBLS9776).

## Supplementary Materials

The following supporting information can be downloaded at: https://www.mdpi.com/article/10.3390/biomedicines12112431/s1, Table S1: List of the quantified signals and their relative assignment and multiplicity; Table S2: Aetiology and clinical characteristics of the AP patients; Table S3: Clinical tests of patients with acute pancreatitis of different severity groups; Table S4: Comparison of biochemical tests for the different aetiologies of acute pancreatitis; Table S5: Clinical tests of patients with acute pancreatitis with biliary aetiology; Table S6: Clinical tests of patients with alcohol-induced AP; Table S7: Comparison of metabolites with different aetiology groups of Acute Pancreatitis; Table S8: Correlation of metabolites with the inverted first dimension of KODAMA. Significantly dysregulated metabolites and lipids are represented in bold; Table S9: Spearman correlation between lipoprotein parameters and inverted first dimension of KODAMA. Significantly dysregulated lipoproteins are represented in bold; Table S10: Comparison of Metabolites and lipids with severity in Acute Pancreatitis. Significantly dysregulated lipids are represented in bold; Table S11: Comparison of metabolites with worse clinical outcomes in Acute Pancreatitis. The ketoacidosis metabolite, 3-hydroxybutyrate, which is significantly dysregulated, as well as acetoacetate and creatinine, are represented in bold; Table S12: Comparison of metabolites with organ dysfunction in acute pancreatitis. Significantly dysregulated lipids and other significantly altered metabolites are represented in bold.
