# Supplementary material for: Metabolites and Lipoproteins May Predict the Severity of Early Acute Pancreatitis in a South African Cohort
Source: Biomedicines. 2024 Oct 23;12(11):2431. doi: 10.3390/biomedicines12112431 (PMC11592248; doi:10.3390/biomedicines12112431)
Supplement: Supplementary file 1 [file biomedicines-12-02431-s001.zip › biomedicines-3209001-supplementary.pdf]

SUPPLEMENTARY TABLES

Table S1. List of the quantified signals and their relative assignment and multiplicity.

Metabolite	Assignment (multiplicity)
Formate	8.45 (s)
Unknown signal at 8.12 ppm	8.12 (d)
Unknown signal at 8.07 ppm	8.07 (d)
Phenylalanine	7.42 (m)
Tyrosine	7.19 (m)
Unknown signal at 7.14 ppm	7.14 (m)
Histidine	7.05 (d)
Glucose	5.23 (d)
Mannose	5.18 (d)
Unknown signal at 5.15 ppm	5.15 (d)
Unknown signal at 5.09 ppm	5.09 (d)
Unknown signal at 5.01 ppm	5.01 (d)
Ascorbate	4.50 (d)
Threonine	4.24 (m)
Lactate	4.11 (q)
Creatinine	4.05 (s)
Creatine	3.92 (s)
Glycine	3.55 (s)
Methanol	3.35 (s)
Unknown signal at 2.55 ppm	2.55 (s)
Citrate	2.53 (d)
Glutamine	2.45 (m)
Pyruvate	2.36 (s)
Glutamate	2.53 (m)
Acetoacetate	2.22 (s)
Acetate	1.91 (s)
Alanine	1.47 (d)
Unknown signal at 1.45 ppm	1.45 (d)
Unknown signal at 1.43 ppm	1.43 (d)
3-Hydroxybutyrate	1.19 (d)
Ethanol	1.17 (t)
Unknown signal at 1.16 ppm	1.16 (d)
Unknown signal at 1.14 ppm	1.14 (d)
Unknown signal at 1.11 ppm	1.11 (d)
Unknown signal at 1.06 ppm	1.06 (d)
Valine	1.04 (d)
Isoleucine	1.00 (d)
Leucine	0.95 (dd)
2-Hydroxybutyrate	0.89 (t)
Protein NH	10.00-6.00
Unsaturated lipid -CH=CH-	5.50-5.10
Lipid alpha-CH2	2.25-2.15
Cholesterol	0.70-0.60
Lipid =CH-CH2-CH=	2.85-2.65
Glycerol phospholipid	4.08-4.03
Phospholipid	3.68-3.62
Lipid beta-CH2	1.65-1.40
Lipid CH2	1.40-1.10
Lipid CH3	1.10-1.08
GlycB	2.07 (m)
GlycA	2.03 (m)

Abbreviations: s, singlet; d, doublet; dd, doublet of doublets; m, multiplet; q, quartet; t, triplet.

Table S2. Aetiology and clinical characteristics of the AP patients

Feature	Biliary (N=12)	Alcohol (N=16)	ARVs (N=2)	p-value
BMI, median [IQR]	39.8 [34.4 43.4]	26.4 [23.7 29.5]	22.6 [22.6 22.6]	0.0134
Age, median [IQR]	47.5 [37.2 58]	36.5 [30.8 44.5]	50 [46 54]	0.0893
Gender				0.0129
Female, n (%)	9 (75.0)	4 (25.0)	2 (100.0)	
Male, n (%)	3 (25.0)	12 (75.0)	0 (0.0)	
Days of hospitalization, median [IQR]	10.5 [8.8 13.5]	8 [4.8 9.5]	7 [4 10]	0.203
Renovascular disease				0.402
No, n (%)	10 (83.3)	14 (87.5)	1 (50.0)	
Yes, n (%)	2 (16.7)	2 (12.5)	1 (50.0)	
Diabetic				<0.001
No, n (%)	12 (100.0)	15 (93.8)	2 (100.0)	
Yes, n (%)	0 (0.0)	1 (6.2)	0 (0.0)	
HIV				0.188
No, n (%)	11 (91.7)	12 (75.0)	1 (50.0)	
Yes, n (%)	1 (8.3)	4 (25.0)	1 (50.0)	
Organ dysfunction				0.250
No, n (%)	8 (66.7)	10 (62.5)	1 (50.0)	
renal, n (%)	0 (0.0)	1 (6.2)	0 (0.0)	
respiratory, n (%)	2 (16.7)	5 (31.2)	0 (0.0)	
transient renal, n (%)	2 (16.7)	0 (0.0)	1 (50.0)	
Local Complications				<0.001
No, n (%)	10 (83.3)	11 (78.6)	2 (100.0)	
Yes, n (%)	2 (16.7)	3 (21.4)	0 (0.0)	
Admission to ICU				0.590
No, n (%)	10 (83.3)	13 (81.2)	1 (50.0)	
Yes, n (%)	2 (16.7)	3 (18.8)	1 (50.0)	
Surgical procedures				0.0481
cholecystectomy, n (%)	3 (27.3)	0 (0.0)	0 (0.0)	
ERCP, n (%)	2 (18.2)	0 (0.0)	0 (0.0)	
None, n (%)	6 (54.5)	14 (100.0)	2 (100.0)	
hospital death				0.291
No, n (%)	11 (91.7)	13 (81.2)	1 (50.0)	
Yes, n (%)	1 (8.3)	3 (18.8)	1 (50.0)	

Abbreviations: ARVs, Antiretrovirals; BMI, Body mass index; HIV, human immunodeficiency virus; ICU, Intensive care unit; ERCP, Endoscopic retrograde cholangiopancreatography; IQR, Interquartile range.

Table S3. Clinical tests of patients with acute pancreatitis of different severity groups. To delineate the changes in acute pancreatitis, the Spearman correlation test was performed to correlate the clinical test values to the different groups in the following rank order: MAP=1, MSAP=2, and SAP=3.

**Features**	**Physiological range**	**MAP (n=8), median**	**MSAP (n=14), median**	**SAP (n=8), median**	***rho***	***p*-value**	**FDR**
Amylase (U/L)	28-110	1009	652	794	0.05	0.820	0.865
Lipase (U/L)	13-60	649	760	1170	0.11	0.640	0.846
WBC (10^9/L)	3.92-10.40	10.8	12.75	8.2	-0.12	0.538	0.846
Haemoglobin (g/dL)	13.4-17.5	12.8	14.35	13.95	0.20	0.328	0.846
Haematocrit, (L/L)	0.390-0.510	0.35	0.4	0.4	0.19	0.142	0.846
Platelets (10^9/L)	171-388	230	227.5	224.5	-0.08	0.668	0.846
Na (mmol/L)	136-145	136	136	137	0.11	0.618	0.846
K (mmol/L)	3.5-5.1	4.2	4.3	4.5	0.15	0.523	0.846
Cl (mmol/L)	98-107	96	100	85	0.17	0.578	0.846
Urea (mmol/L)	2.1-7.1	4	8.75	9.6	0.27	0.364	0.846
CRP (mg/L)	<10	83	115	232	0.06	0.793	0.865
Total protein (g/L)	60-78	71.5	69	62	-0.27	0.391	0.846
Albumin (g/L)	35-52	37	35.5	34.5	-0.18	0.391	0.846
Total bilirubin (µmol/L)	5-21	20	17.5	48.5	0.18	0.400	0.846
Conjugated bilirubin (µmol/L)	0-3	12	5.5	31.5	0.15	0.468	0.846
ALT (U/L)	10-40	34	33.5	102.5	0.14	0.509	0.846
AST (U/L)	15-40	35	36	164	0.33	0.102	0.846
ALP (U/L)	53-128	91	111	144	0.03	0.901	0.901
GGT(U/L)	< 68	375	119	397	0.07	0.749	0.865

Abbreviations: ALP, Alkaline phosphatase; ALT, Alanine transaminase; AST, Aspartate transaminase; CRP, C-reactive protein; FDR, False discovery rate; GGT, Gamma glutamyl transferase; MAP, mild acute pancreatitis; MSAP, moderate severe acute pancreatitis; SAP, severe acute pancreatitis; WBC, White blood count.

Table S4. Comparison of biochemical tests for the different aetiologies of acute pancreatitis

Feature	Biliary (n=12), median [IQR]	Alcohol (n=16), median [IQR]	ARVs (n=2), median [IQR]	p-value	FDR
Amylase (U/L)	1914 [1696.5 2642]	450 [370.5 794]	450 [450 450]	0.001	0.008
Lipase (U/L)	1129.5 [529.5 1678.25]	749 [489.75 1031]	NA	0.339	0.496
WBC (10^9/L)	13.1 [10.9 14.7]	8.05 [6.875 10.875]	12.1 [8.95 15.25]	0.032	0.154
Haemoglobin (g/dL)	13.4 [11.2 14.8]	13.95 [12.675 15.25]	12.55 [11.525 13.575]	0.733	0.871
Haematocrit (L/L)	0.4 [0.325 0.475]	0.4 [0.35 0.4]	0.4 [0.35 0.45]	0.914	0.961
Platelets (10^9/L)	235 [212 303.5]	209.5 [133.75 252.75]	264 [256 272]	0.183	0.387
Na (mmol/L)	140 [135 140]	134.5 [129 137]	137 [137 137]	0.240	0.432
K (mmol/L)	4.4 [4 27.375]	4.4 [3.95 5.175]	3.2 [3.2 3.2]	0.325	0.496
Cl (mmol/L)	54 [29.2 83.5]	100 [96 101]	97 [97 97]	0.570	0.774
Urea (mmol/L)	7.5 [5.8 8.8]	8.5 [2.9 12.45]	8.7 [8.7 8.7]	0.961	0.961
CRP (mg/L)	46 [32 136]	162.5 [70.25 297.25]	337 [337 337]	0.145	0.344
Total Protein (g/L)	66.5 [60.75 71.25]	66 [57.5 72]	62 [62 62]	0.908	0.961
Albumin (g/L)	36 [35 38.5]	35 [31 41]	29.5 [24.75 34.25]	0.715	0.871
Total bilirubin (μmol/L)	42.5 [15.5 91.75]	15 [8 21]	31 [22 40]	0.250	0.432
Conjugated bilirubin (μmol/L)	21.5 [11.25 57.5]	5 [4 6]	26 [16.5 35.5]	0.077	0.209
ALT (U/L)	160 [82.5 232]	23 [22 37]	27 [22 32]	0.013	0.085
AST (U/L)	91.5 [44.25 231.75]	33 [27.25 46.25]	50 [42 58]	0.074	0.209
ALP (U/L)	267 [231.25 304]	75 [64.25 90.25]	93.5 [84.75 102.25]	<0.001	0.003
GGT (U/L)	468 [328.5 1028.25]	175.5 [65.25 317.75]	192 [117.5 266.5]	0.053	0.200

Abbreviations: ALP, Alkaline phosphatase; ALT, Alanine transaminase; AST, Aspartate transaminase; CRP, C-reactive protein; FDR, False discovery rate; GGT, Gamma glutamyl transferase; IQR, Interquartile range; WBC, White blood count.

Table S5. Clinical tests of patients with acute pancreatitis with biliary aetiology. To delineate the changes in acute pancreatitis, the Spearman correlation test was performed to correlate the clinical test values to the different groups in the following rank order: MAP=1, MSAP=2, and SAP=3.

Feature	MAP (n=4), median [IQR]	MSAP (n=5), median [IQR]	SAP (n=3) , median [IQR]	rho	p-value	FDR
Amylase (U/L)	1710.5 [1486.5 2193.75]	2604 [1993.5 3840.75]	1914 [1205 2038.5]	0.05	0.877	0.987
Lipase (U/L)	1294 [797 1791]	847.5 [471.25 1566.75]	1322.5 [1246.25 1398.75]	0.15	0.715	0.933
WBC (10^9/L)	11 [10.9 13.5]	14 [13.1 15.2]	8.4 [8.2 11.3]	-0.23	0.489	0.909
Haemoglobin (g/dL)	10 [10 10]	13.4 [11.2 14.2]	16.1 [14.75 16.25]	0.71	0.031	0.325
Haematocrit, (L/L)	0.34 [0.337 0.344]	0.408 [0.355 0.443]	0.45 [0.414 0.496]	0.66	0.036	0.325
Platelets (10^9/L)	323 [276.5 332.5]	242 [210 284]	214 [158 224.5]	-0.51	0.112	0.505
Na (mmol/L)	140 [71.75 142]	139 [135 139.5]	140 [140 140]	-0.04	0.932	0980
K (mmol/L)	35 [18.95 71]	4.1 [4 4.2]	4.5 [4.5 4.5]	-0.19	0.725	0.933
Cl (mmol/L)	-	-	-	-	-	-
Urea (mmol/L)	-	-	-	-	-	-
Creatinine (mg/dL)	53.5 [50.75 56.25]	107 [87.5 113.75]	182 [139.5 224.5]	0.54	0.167	0.516
CRP (mg/L)	60 [48.5 71.5]	46 [32 136]	123.5 [69.25 177.75]	0		
Total protein (g/L)	72 [72 72]	65.5 [59.5 78.5]	62 [61.5 66.5]	-0.26	0.541	0.909
Albumin (g/L)	38 [37.5 38.5]	35 [35 36]	36 [34.5 38]	-0.21	0.556	0.909
Total bilirubin (µmol/L)	35 [27.5 42.5]	14 [9 35]	115 [95.5 117.5]	0.55	0.102	0.505
Conjugated bilirubin (µmol/L)	21.5 [18.25 24.75]	11 [2 12]	58 [57 77]	0.45	0.193	0.516
ALT (U/L)	208 [200.5 215.5]	75 [38 127]	235 [170 316.5]	0.14	0.690	0.933
AST (U/L)	88 [78.5 97.5]	36 [30 76]	239 [224.5 248.5]	0.44	0.201	0.516
ALP (U/L)	335 [300 370]	225 [187 269]	271 [260.5 293]	-0.05	0.885	0.987
GGT (U/L)	956 [665.5 1246.5]	313 [119 426]	1187 [848.5 1472]	0.3	0.395	0.889

Abbreviations: ALP, Alkaline phosphatase; ALT, Alanine transaminase; AST, Aspartate transaminase; CRP, C-reactive protein; FDR, False discovery rate; GGT, Gamma glutamyl transferase; IQR, Interquartile range; MAP, mild acute pancreatitis; MSAP, moderate severe acute pancreatitis; SAP, severe acute pancreatitis; WBC, White blood count.

Table S6. Clinical tests of patients with alcohol-induced AP. To delineate the changes in acute pancreatitis, the Spearman correlation test was performed to correlate the clinical test values to the different groups in the following rank order: MAP=1, MSAP=2, and SAP=3.

**Feature**	**MAP (n=4), median [IQR]**	**MSAP (n=8), median [IQR]**	**SAP (n=4), median [IQR]**	**rho**	**p-value**	**FDR**
Amylase (U/L)	390 [265 620]	418 [372 798.75]	764 [566.5 794]	0.21	0.460	0.932
Lipase (U/L)	649 [379.25 947.5]	760 [686.5 926.5]	738 [402 1068.5]	-0.01	0.974	0.974
WBC (10x^9/L)	6.55 [4.625 9.85]	9.15 [7.1 11.35]	8.95 [7.575 10.5]	0.15	0.570	0.932
Haemoglobin (g/dL)	13.25 [12.675 13.875]	15.3 [14.1 15.45]	12.7 [11.275 13.925]	-0.12	0.671	0.932
Haematocrit, (L/L)	0.408 [0.374 0.433]	0.438 [0.412 0.459]	0.381 [0.342 0.417]	-0.18	0.521	0.932
Platelets (10x^9/L)	200 [177.5 219.5]	180.5 [120.25 258]	224 [187.75 279.25]	0.13	0.620	0.932
Na (mmol/L)	132 [99.225 136.25]	134.5 [132.5 136.5]	134.5 [100.15 138.25]	0.13	0.659	0.932
K (mmol/L)	4.15 [3.975 4.25]	5.05 [4.05 5.375]	4.45 [4.275 22.625]	0.35	0.217	0.932
Cl (mmol/L)	96.5 [96.25 96.75]	100 [95.75 102.75]	101 [85 319.5]	0.29	0.455	0.932
Urea (mmol/L)	6.25 [5.125 7.375]	9.2 [5.35 34.6]	8.75 [5.275 12.225]	0.09	0.840	0.932
Creatinine (mg/dL)	99.5 [81 112.5]	65 [64 134]	75.5 [69.25 252]	-0.05	0.865	0.932
CRP (mg/L)	191.5 [78.75 318.5]	119 [46.75 247.5]	222 [155.75 261.75]	-0.05	0.874	0.932
Total protein (g/L)	71 [62 71.5]	69.5 [61 72]	61 [53.5 63]	-0.28	0.387	0.932
Albumin (g/L)	35 [33 39.5]	40.5 [34 42.5]	31 [29.5 33.5]	-0.33	0.264	0.932
Total bilirubin (µmol/L)	11 [10 16]	17.5 [9.75 20.75]	14 [6.5 47.75]	-0.04	0.885	0.932
Conjugated bilirubin (µmol/L)	5 [4.5 8.5]	4.5 [4 5.75]	5 [3.5 33.5]	-0.05	0.868	0.932
ALT (U/L)	23 [22.5 28.5]	22 [19.5 28.25]	68.5 [33.25 118.5]	0.37	0.217	0.932
AST (U/L)	29 [26.5 32]	31 [27.5 42]	79.5 [33.5 164.75]	0.37	0.199	0.932
ALP (U/L)	88 [87.5 89.5]	64 [56.5 66]	104 [75 138.5]	0.11	0.708	0.932
GGT (U/L)	319 [181.5 347.5]	81 [57.5 203]	271.5 [216.5 356]	0.1	0.733	0.932

Abbreviations: ALP, Alkaline phosphatase; ALT, Alanine transaminase; AST, Aspartate transaminase; CRP, C-reactive protein; FDR, False discovery rate; GGT, Gamma glutamyl transferase; IQR, Interquartile range; MAP, mild acute pancreatitis; MSAP, moderate severe acute pancreatitis; SAP, severe acute pancreatitis; WBC, White blood count.

Table S7: Comparison of metabolites with different aetiology groups of Acute Pancreatitis.

**Metabolites**	**Biliary (n12), median [IQR]**	**Alcohol (n=16), median [IQR]**	**ARVs (n=2), median [IQR]**	**p-value**	**FDR**
Formate	0.016 [0.012 0.02]	0.015 [0.01 0.017]	0.01 [0.009 0.011]	0.462	0.760
Unknown signal at 8.12 ppm	0.006 [0 0.012]	0.003 [0 0.009]	0.006 [0.003 0.008]	0.811	0.871
Unknown signal at 8.07 ppm	0 [0 0]	0 [0 0.001]	0 [0 0]	0.730	0.871
Phenylalanine	0.147 [0.106 0.158]	0.129 [0.121 0.15]	0.279 [0.218 0.341]	0.218	0.760
**Tyrosine**	**0.037 [0.033 0.04]**	**0.054 [0.045 0.058]**	**0.049 [0.046 0.051]**	**0.005**	**0.203**
Unknown signal at 7.14 ppm	0.007 [0 0.041]	0 [0 0.024]	0.019 [0.009 0.028]	0.806	0.871
Histidine	0.088 [0.065 0.095]	0.073 [0.063 0.083]	0.096 [0.085 0.108]	0.522	0.760
Glucose	3.138 [2.846 4.186]	3.399 [2.324 3.909]	2.53 [2.451 2.609]	0.348	0.760
Mannose	0.058 [0.045 0.065]	0.066 [0.047 0.098]	0.082 [0.071 0.093]	0.398	0.760
Unknown signal at 5.15 ppm	0 [0 0]	0 [0 0]	0 [0 0]	0.472	0.760
Unknown signal at 5.09 ppm	0 [0 0.025]	0 [0 0]	0.011 [0.006 0.017]	0.351	0.760
Unknown signal at 5.01 ppm	0 [0 0]	0 [0 0]	0 [0 0]	0.472	0.760
Ascorbate	0 [0 0.002]	0 [0 0.01]	0 [0 0]	0.516	760
Threonine	0.072 [0.045 0.096]	0.061 [0.03 0.076]	0 [0 0]	0.126	0.584
Lactate	2.861 [2.367 4.054]	3.022 [2.291 3.63]	3.811 [3.289 4.334]	0.707	0.871
Creatinine	0.072 [0.063 0.089]	0.072 [0.054 0.1]	0.175 [0.137 0.213]	0.261	0.760
Creatine	0.032 [0.012 0.07]	0.058 [0.02 0.11]	0.138 [0.075 0.2]	0.633	0.852
Glycine	0.312 [0.27 0.354]	0.271 [0.227 0.31]	0.335 [0.333 0.338]	0.273	0.760
Methanol	0.067 [0.035 0.101]	0.039 [0.025 0.051]	0.041 [0.04 0.043]	0.348	0.760
Unknown signal at 2.55 ppm	0 [0 0]	0 [0 0]	0.032 [0.023 0.04]	0.009	0.203
Citrate	0.09 [0.046 0.121]	0.07 [0.062 0.084]	0 [0 0]	0.107	0.561
Glutamine	0.269 [0.249 0.284]	0.29 [0.239 0.329]	0.306 [0.293 0.32]	0.617	0.852
Pyruvate	0.064 [0.044 0.094]	0.064 [0.038 0.106]	0.161 [0.14 0.181]	0.144	0.611
Glutamate	0.166 [0.121 0.209]	0.169 [0.152 0.211]	0.198 [0.197 0.199]	0.652	0.852
**Acetoacetate**	**0.073 [0.04 0.112]**	**0.193 [0.116 0.415]**	**0.182 [0.119 0.244]**	**0.012**	**0.203**
Acetate	0.032 [0.024 0.034]	0.028 [0.024 0.033]	0.033 [0.032 0.033]	0.837	0.871
Alanine	0.355 [0.32 0.434]	0.341 [0.307 0.365]	0.42 [0.399 0.441]	0.417	0.760
Unknown signal at 1.45 ppm	0.053 [0.044 0.065]	0.056 [0.041 0.079]	0.074 [0.059 0.089]	0.785	0.871
Unknown signal at 1.43 ppm	0 [0 0]	0 [0 0]	0 [0 0]	0.472	0.760
**3-Hydroxybutyrate**	**0.086 [0.051 0.203]**	**0.435 [0.13 1.692]**	**0.244 [0.164 0.324]**	**0.055**	**0.478**
Ethanol	0.006 [0 0.014]	0 [0 0]	0.038 [0.019 0.058]	0.110	0.561
Unknown signal at 1.16 ppm	0.039 [0 0.096]	0.126 [0 5.133]	0.767 [0.384 1.151]	0.514	0.760
Unknown signal at 1.14 ppm	0.022 [0 0.039]	0 [0 0.021]	0.148 [0.106 0.191]	0.075	0.478
Unknown signal at 1.11 ppm	0.077 [0.059 0.083]	0.086 [0.073 0.104]	0.07 [0.066 0.074]	0.157	0.617
Unknown signal at 1.06 ppm	0.042 [0.032 0.05]	0.053 [0.036 0.094]	0.064 [0.051 0.076]	0.377	0.760
**Valine**	**0.178 [0.155 0.218]**	**0.237 [0.21 0.268]**	**0.236 [0.206 0.265]**	**0.059**	**0.478**
Isoleucine	0.038 [0.027 0.054]	0.059 [0.05 0.068]	0.072 [0.064 0.08]	0.420	0.478
Leucine	0.072 [0.062 0.099]	0.099 [0.083 0.11]	0.104 [0.094 0.115]	0.066	0.478
2-Hydroxybutyrate	0.011 [0 0.079]	0.072 [0.02 0.099]	0.062 [0.038 0.085]	0.367	0.760
Protein NH	133.86 [119.887 139.57]	130.264 [119.303 139.803]	149.873 [138.618 161.129]	0.701	0.871
Unsaturated lipid -CH=CH-	10.76 [9.163 13.55]	9.782 [8.757 13.015]	10.199 [10.137 10.261]	0.955	0.955
Lipid alpha-CH2	1.808 [1.095 3.333]	2.19 [1.73 2.879]	1.573 [1.534 1.613]	0.517	0.760
Cholesterol	0.911 [0.514 1.015]	0.639 [0.586 0.734]	0.843 [0.768 0.918]	0.234	0.760
Lipid =CH-CH2-CH=	5.073 [3.569 5.666]	4.309 [3.946 5.495]	4.826 [4.748 4.903]	0.821	0.871
Glycorol phospholipid	0.416 [0.122 0.978]	0.504 [0.249 0.848]	0.18 [0.098 0.262]	0.648	0.852
Phospholipid	3.675 [3.353 4.714]	3.602 [3.346 4.345]	4.881 [4.482 5.281]	0.362	0.760
Lipid beta-CH2	6.904 [6.076 8.183]	5.872 [5.297 8.456]	6.659 [6.554 6.764]	0.737	0.871
Lipid CH2	64.754 [50.366 75.038]	62.248 [53.163 83.574]	59.558 [58.656 60.461]	0.881	0.899
Lipid CH3	25.489 [21.931 29.467]	23.373 [19.958 27.419]	25.432 [25.227 25.637]	70.78	0.871
GlycB	0.374 [0.364 0.48]	0.412 [0.367 0.467]	0.56 [0.505 0.614]	0.286	0.760
GlycA	1.719 [1.503 2.005]	1.913 [1.59 2.185]	2.62 [2.287 2.953]	0.316	0.760

Abbreviations: ARVs, antiretrovirals; FDR, False discovery rate; IQR, Interquartile range

Table S8. Correlation of metabolites with inverted first dimension of KODAMA. Significantly dysregulated metabolites and lipids are represented in bold.

Feature	rho	p-value	FDR
Formate	-0.05	0.776	0.842
Unknown signal at 8.12 ppm	-0.11	0.531	0.639
Unknown signal at 8.07 ppm	0.05	0.747	0.828
**Phenylalanine**	**-0.51**	**0.001**	**0.005**
Tyrosine	0.26	0.116	0.211
Unknown signal at 7.14 ppm	-0.31	0.062	0.138
Histidine	0.19	0.266	0.357
Glucose	-0.04	0.834	0.868
**Mannose**	**-0.46**	**0.005**	**0.017**
Unknown signal at 5.15 ppm	-0.28	0.092	0.174
Unknown signal at 5.09 ppm	-0.17	0.306	0.401
Unknown signal at 5.01 ppm	-0.28	0.092	0.174
**Ascorbate**	**0.44**	**0.006**	**0.020**
**Threonine**	**0.35**	**0.032**	**0.0898**
**Lactate**	**-0.62**	**<0.001**	**<0.001**
Creatinine	0.01	0.934	0.934
Creatine	-0.31	0.062	0.138
Glycine	0.02	0.928	0.934
**Methanol**	**0.39**	**0.018**	**0.053**
Unknown signal at 2.55 ppm	-0.2	0.229	0.341
Citrate	0.2	0.242	0.341
**Glutamine**	**0.57**	**<0.001**	**0.002**
**Pyruvate**	**-0.33**	**0.046**	**0.110**
Glutamate	-0.16	0.329	0.420
**Acetoacetate**	**-0.53**	**<0.001**	**0.004**
Acetate	-0.13	0.425	0.529
Alanine	0.21	0.216	0.333
Unknown signal at 1.45 ppm	-0.45	0.005	0.018
Unknown signal at 1.43 ppm	-0.28	0.092	0.174
**3-Hydroxybutyrate**	**-0.35**	**0.034**	**0.090**
**Ethanol**	**0.63**	**<0.001**	**<0.001**
Unknown signal at 1.16 ppm	-0.1	0.566	0.656
Unknown signal at 1.14 ppm	-0.2	0.242	0.341
Unknown signal at 1.11 ppm	-0.52	<0.000	0.004
Unknown signal at 1.06 ppm	-0.33	0.044	0.110
Valine	0.22	0.200	0.320
Isoleucine	0.04	0.834	0.868
Leucine	-0.1	0.538	0.639
**2-Hydroxybutyrate**	**-0.52**	**<0.001**	**0.004**
**Protein NH**	**0.82**	**<0.001**	**<0.001**
Unsaturated lipid -CH=CH-	0.65	0.000	0.000
Lipid alpha-CH_2	-0.28	0.089	0.174
**Cholesterol**	**0.62**	**<0.001**	**<0.001**
**Lipid =CH-CH_2-CH=**	**0.74**	**<0.001**	**<0.001**
Glycorol phospholipid	0.07	0.695	0.788
Phospholipid	0.22	0.199	0.320
**Lipid beta-CH_2**	**0.77**	**<0.001**	**<0.001**
Lipid CH_2	0.19	0.247	0.341
**Lipid CH3**	**0.81**	**<0.001**	**<0.001**
GlycB	-0.23	0.176	0.309
GlycA	-0.22	0.189	0.320

Abbreviations: FDR, False discovery rate

Table S9. Spearman correlation between lipoprotein parameters and inverted first dimension of KODAMA. Significantly dysregulated lipoproteins are represented in bold.

Feature	rho	p-value	FDR
VLDL-C	0.26	0.116	0.216
**IDL-C**	**0.32**	**0.050**	**0.131**
**LDL-C**	**-0.39**	**0.018**	**0.059**
**HDL-C**	**-0.63**	**<0.001**	**<0.001**
VLDL-TG	0.23	0.171	0.278
IDL-TG	0.26	0.114	0.216
LDL-TG	0.21	0.204	0.294
HDL-TG	0.22	0.185	0.283
VLDL-P (nmol/L)	0.25	0.135	0.234
Large VLDL-P (nmol/L)	0.26	0.113	0.216
Medium VLDL-P (nmol/L)	0.14	0.409	0.466
Small VLDL-P (nmol/L)	0.27	0.111	0.216
**LDL-P (nmol/L)**	**-0.4**	**0.015**	**0.059**
Large LDL-P (nmol/L)	-0.18	0.283	0.369
Medium LDL-P (nmol/L)	-0.18	0.274	0.369
**Small LDL-P (nmol/L)**	**-0.47**	**0.004**	**0.019**
**HDL-P (µmol/L)**	**-0.65**	**<0.001**	**<0.001**
Large HDL-P (µmol/L)	0.02	0.895	0.895
Medium HDL-P (µmol/L)	-0.08	0.650	0.676
**Small HDL-P (µmol/L)**	**-0.68**	**<0.001**	**<0.001**
VLDL-Z (nm)	0.1	0.542	0.587
**LDL-Z (nm)**	**0.34**	**0.0392**	**0.113**
**HDL-Z (nm)**	**0.68**	**<0.001**	**<0.001**
**Non-HDL-P (nmol/L)**	**-0.4**	**0.016**	**0.059**
Total-P/HDL-P	0.16	0.338	0.418
LDL-P/HDL-P	0.14	0.412	0.466

Abbreviations: FDR, False discovery rate; HDL, High-density lipoprotein; LDL, Low-density lipoprotein; IDL, Intermediate density lipoproteins; VLDL, Very Low-Density Lipoprotein; TG, Triglyceride; C, Cholesterol; Z, size; P, particle.

Table S10. Comparison of metabolic changes with severity in Acute Pancreatitis over time. The changes were calculated as the difference in concentration between two time points divided by the number of days in between. Significantly dysregulated lipids are represented in bold.

Feature	MAP, median [IQR]	MSAP, median [IQR]	SAP, median [IQR]	p-value	FDR
Formate	0.001 [-0.001 0.002]	0.004 [0.002 0.007]	0.002 [-0.002 0.004]	0.288	0.538
Unknown signal at 8.12 ppm	0.001 [-0.003 0.005]	0.001 [0 0.003]	0.004 [-0.001 0.008]	0.673	0.838
Unknown signal at 8.07 ppm	0 [0 0]	0 [0 0.003]	0.002 [0 0.003]	0.661	0.838
Phenylalanine	-0.01 [-0.015 0.004]	0.004 [-0.01 0.015]	0.014 [0.008 0.018]	0.033	0.212
Tyrosine	0.002 [-0.002 0.006]	0 [-0.007 0.003]	-0.001 [-0.003 0.004]	0.327	0.576
Unknown signal at 7.14 ppm	0.021 [0 0.024]	0 [-0.001 0.015]	-0.003 [-0.009 0]	0.071	0.248
Histidine	-0.005 [-0.009 0.003]	-0.001 [-0.008 0.011]	0.001 [-0.004 0.005]	0.491	0.716
Glucose	-0.015 [-0.113 0.461]	0.133 [-0.042 0.476]	-0.425 [-0.693 0.155]	0.466	0.710
Mannose	-0.002 [-0.013 0.007]	0.002 [-0.01 0.004]	0.001 [-0.007 0.012]	0.801	0.923
Unknown signal at 5.15 ppm	0 [0 0]	0 [0 0]	0 [0 0]	0.276	0.538
Unknown signal at 5.09 ppm	0.001 [0 0.013]	0 [-0.001 0.004]	0 [0 0.004]	0.548	0.740
Unknown signal at 5.01 ppm	0 [0 0]	0 [0 0]	0 [0 0]	0.739	0.898
Ascorbate	0 [0 0.004]	0 [0 0.003]	-0.004 [-0.005 0]	0.115	0.311
Threonine	0.007 [0.003 0.028]	-0.003 [-0.008 0.009]	0.004 [-0.012 0.029]	0.473	0.710
Lactate	-0.001 [-0.194 0.312]	0.213 [-0.064 0.42]	0.009 [-0.766 0.068]	0.220	0.487
Creatinine	-0.001 [-0.005 0.006]	0.001 [-0.002 0.006]	-0.011 [-0.083 0.001]	0.204	0.473
Creatine	0 [-0.004 0.001]	-0.011 [-0.026 0]	-0.004 [-0.036 -0.003]	0.241	0.512
Glycine	0.01 [-0.001 0.02]	0.034 [0.001 0.042]	0.026 [0.002 0.041]	0.424	0.697
Methanol	0.003 [-0.011 0.012]	0.002 [-0.004 0.036]	0.001 [-0.002 0.001]	0.393	0.668
Unknown signal at 2.55 ppm	0 [0 0]	0 [0 0.006]	0 [0 0.036]	0.263	0.537
Citrate	-0.003 [-0.011 0.006]	-0.001 [-0.024 0.006]	-0.002 [-0.014 0.005]	0.890	0.923
Glutamine	-0.013 [-0.039 0]	0.026 [0.003 0.05]	0.028 [0.012 0.032]	0.098	0.311
Pyruvate	-0.008 [-0.022 0]	0.004 [-0.014 0.013]	0.005 [-0.013 0.016]	0.470	0.710
Glutamate	0.02 [0.003 0.046]	0.034 [0.018 0.063]	0.03 [-0.01 0.052]	0.829	0.923
Acetoacetate	0.003 [-0.007 0.007]	-0.022 [-0.067 0.031]	0.145 [0.061 0.421]	0.007	0.092
Acetate	-0.001 [-0.003 0.007]	0.002 [-0.001 0.006]	0.004 [-0.001 0.007]	0.819	0.923
Alanine	0.006 [-0.036 0.046]	0.02 [-0.009 0.044]	0.012 [-0.023 0.027]	0.876	0.923
Unknown signal at 1.45 ppm	0 [-0.005 0]	0.005 [-0.001 0.012]	0.004 [-0.007 0.017]	0.295	0.538
Unknown signal at 1.43 ppm	0 [0 0]	0 [0 0]	0 [0 0]	1.000	1.000
3-Hydroxybutyrate	0.002 [-0.015 0.003]	-0.023 [-0.213 0.016]	0.514 [0.187 0.781]	0.012	0.092
Ethanol	0.003 [-0.003 0.008]	0 [0 0]	0 [0 0.009]	0.623	0.814
Unknown signal at 1.16 ppm	-0.003 [-0.683 0.009]	-0.012 [-0.058 0.012]	0 [-0.448 0]	0.866	0.923
Unknown signal at 1.14 ppm	0 [-0.003 0.012]	-0.007 [-0.019 0.012]	0 [0 0.015]	0.520	0.737
Unknown signal at 1.11 ppm	-0.01 [-0.013 -0.005]	-0.002 [-0.01 0.003]	-0.002 [-0.004 0.004]	0.162	0.393
Unknown signal at 1.06 ppm	-0.004 [-0.005 -0.001]	-0.007 [-0.012 0]	0.007 [0.004 0.015]	0.004	0.092
Valine	-0.009 [-0.027 0.001]	0.002 [-0.009 0.024]	0.02 [0.011 0.054]	0.071	0.248
Isoleucine	0.003 [-0.005 0.009]	0 [-0.002 0.012]	0.01 [0.002 0.013]	0.551	0.740
Leucine	-0.006 [-0.011 0.001]	0.001 [-0.005 0.011]	0.01 [0.006 0.018]	0.073	0.248
2-Hydroxybutyrate	-0.009 [-0.013 -0.003]	-0.005 [-0.025 0]	0.004 [-0.005 0.036]	0.116	0.311
Protein NH	1.652 [-5.597 3.752]	1.062 [-6.484 14.877]	-2.663 [-5.193 3.942]	0.905	0.923
Unsaturated lipid -CH=CH-	-0.226 [-0.746 -0.077]	0.12 [-0.457 1.535]	0.905 [0.578 1.35]	0.108	0.311
Lipid alpha-CH_2	-0.275 [-0.414 0.046]	0.018 [-0.128 0.213]	0.344 [-0.118 0.564]	0.148	0.377
**Cholesterol**	**-0.026 [-0.06 0.003]**	**0.02 [-0.052 0.106]**	**0.054 [0.044 0.176]**	**0.013**	**0.092**
**Lipid =CH-CH_2-CH=**	**-0.121 [-0.286 -0.01]**	**0.093 [-0.105 0.623]**	**0.469 [0.205 0.787]**	**0.013**	**0.092**
Glycorol phospholipid	-0.082 [-0.137 0.003]	-0.002 [-0.214 0.036]	0 [-0.201 0.102]	0.840	0.923
Phospholipid	-0.202 [-0.358 0.008]	0.168 [-0.006 0.267]	0.06 [-0.027 0.146]	0.064	0.248
Lipid beta-CH_2	-0.138 [-0.426 -0.06]	-0.056 [-0.368 0.668]	0.453 [0.206 1.324]	0.061	0.248
Lipid CH_2	-2.675 [-5.831 -1.865]	1.226 [-2.266 7.464]	3.196 [1.422 7.154]	0.068	0.248
**Lipid CH_3**	**-0.014 [-1.985 0.337]**	**0.589 [-0.394 2.7]**	**1.528 [0.599 1.627]**	**0.047**	**0.248**
**GlycB**	**0 [-0.004 0.008]**	**0.045 [0.037 0.089]**	**0.014 [-0.001 0.038]**	**0.010**	**0.092**
**GlycA**	**-0.021 [-0.069 0.039]**	**0.24 [0.149 0.337]**	**0.01 [-0.057 0.12]**	**0.004**	**0.092**

Abbreviations: FDR, False discovery rate; IQR, Interquartile range

Table S11. Comparison of metabolic changes over time with worsening clinical outcomes (*i.e.*, in-hospital death or admission to the ICU) in Acute Pancreatitis. The changes were calculated as the difference in concentration between two time points divided by the number of days in between. The ketoacidosis metabolite, 3-hydroxybutyrate which is significantly dysregulated as well as acetoacetate and creatinine are represented in bold.

**Feature**	**mild outcome, median [IQR]**	**severe outcome, median [IQR]**	**p-value**	**FDR**
Formate	0.003 [0 0.004]	-0.001 [-0.003 0.004]	0.659	0.997
Unknown signal at 8.12 ppm	0.001 [-0.001 0.004]	0.007 [-0.001 0.009]	0.395	0.821
Unknown signal at 8.07 ppm	0 [0 0.004]	0 [0 0.002]	0.798	0.997
Phenylalanine	0.003 [-0.013 0.014]	0.01 [0.006 0.014]	0.325	0.821
Tyrosine	0.001 [-0.003 0.004]	-0.001 [-0.003 0.005]	0.973	1.000
Unknown signal at 7.14 ppm	0 [0 0.021]	-0.006 [-0.012 -0.003]	0.019	0.238
Histidine	-0.003 [-0.008 0.004]	0.001 [-0.006 0.005]	0.812	0.997
Glucose	0.068 [-0.113 0.665]	-0.425 [-0.48 -0.045]	0.164	0.706
Mannose	0.002 [-0.011 0.009]	-0.006 [-0.007 0.001]	0.709	0.997
Unknown signal at 5.15 ppm	0 [0 0]	0 [0 0]	0.057	0.450
Unknown signal at 5.09 ppm	0 [0 0.009]	0 [0 0]	0.414	0.821
Unknown signal at 5.01 ppm	0 [0 0]	0 [0 0]	0.904	0.997
Ascorbate	0 [0 0.004]	-0.004 [-0.005 0]	0.166	0.706
Threonine	0.003 [-0.006 0.023]	0.004 [0 0.016]	0.919	0.997
Lactate	0.151 [-0.193 0.375]	0.009 [-0.719 0.046]	0.126	0.644
**Creatinine**	**0.001 [-0.005 0.007]**	**-0.025 [-0.141 -0.004]**	**0.053**	**0.450**
Creatine	-0.003 [-0.015 0.001]	-0.022 [-0.05 -0.003]	0.262	0.785
Glycine	0.022 [-0.001 0.04]	0.026 [-0.021 0.026]	0.292	0.785
Methanol	0.002 [-0.001 0.018]	-0.002 [-0.002 0.001]	0.096	0.544
Unknown signal at 2.55 ppm	0 [0 0]	0 [0 0]	0.732	0.997
Citrate	-0.001 [-0.02 0.008]	-0.002 [-0.008 0.002]	0.709	0.997
Glutamine	0.012 [-0.019 0.04]	0.013 [0.012 0.028]	1.000	1.000
Pyruvate	-0.001 [-0.018 0.011]	0.005 [-0.016 0.007]	0.919	0.997
Glutamate	0.034 [0.013 0.062]	0.015 [-0.036 0.03]	0.209	0.710
**Acetoacetate**	**0.002 [-0.032 0.018]**	**0.145 [0.074 0.259]**	**0.004**	**0.123**
Acetate	0.002 [-0.002 0.007]	0.002 [-0.005 0.008]	0.865	0.997
Alanine	0.012 [-0.02 0.05]	0.012 [-0.03 0.026]	0.519	0.879
Unknown signal at 1.45 ppm	0 [-0.004 0.009]	0.004 [-0.008 0.012]	0.919	0.997
Unknown signal at 1.43 ppm	0 [0 0]	0 [0 0]	1.000	1.000
**3-Hydroxybutyrate**	**0.002 [-0.055 0.009]**	**0.514 [0.231 0.628]**	**0.003**	**0.123**
Ethanol	0 [0 0.007]	0 [0 0]	0.692	0.997
Unknown signal at 1.16 ppm	0 [-0.063 0.011]	0 [-0.895 0]	0.534	0.879
Unknown signal at 1.14 ppm	0 [-0.014 0.014]	0 [-0.001 0]	0.919	0.997
Unknown signal at 1.11 ppm	-0.004 [-0.012 0.001]	-0.003 [-0.005 -0.002]	0.973	1.000
Unknown signal at 1.06 ppm	-0.004 [-0.007 0.003]	0.007 [0.004 0.009]	0.011	0.184
Valine	-0.003 [-0.015 0.028]	0.017 [0.005 0.02]	0.435	0.821
Isoleucine	0 [-0.005 0.011]	0.01 [0.003 0.012]	0.209	0.710
Leucine	0 [-0.007 0.012]	0.008 [0.003 0.01]	0.359	0.821
2-Hydroxybutyrate	-0.006 [-0.02 0]	-0.003 [-0.007 0.004]	0.395	0.821
Protein NH	2.231 [-6.081 10.547]	-3.854 [-6.531 -2.663]	0.209	0.710
Unsaturated lipid -CH=CH-	-0.154 [-0.404 1.137]	0.667 [0.489 0.905]	0.519	0.879
Lipid alpha-CH_2	-0.03 [-0.286 0.1]	0.344 [-0.166 0.492]	0.476	0.866
Cholesterol	0.005 [-0.052 0.066]	0.049 [0.038 0.148]	0.083	0.530
Lipid =CH-CH_2-CH=	-0.01 [-0.139 0.513]	0.232 [0.177 0.469]	0.292	0.785
Glycorol phospholipid	-0.028 [-0.139 0.02]	0 [-0.332 0.051]	0.865	0.997
Phospholipid	0.031 [-0.164 0.214]	0.06 [-0.077 0.061]	0.760	0.997
Lipid beta-CH_2	-0.111 [-0.267 0.472]	0.386 [0.025 0.453]	0.396	0.821
Lipid CH_2	-0.403 [-3.014 3.979]	2.495 [0.349 4.314]	0.564	0.898
Lipid CH_3	0.366 [-0.619 1.49]	0.679 [0.518 1.528]	0.435	0.821
GlycB	0.035 [0.002 0.049]	0.014 [-0.015 0.026]	0.262	0.785
GlycA	0.126 [0.009 0.267]	-0.045 [-0.068 0.01]	0.062	0.450

Abbreviations: FDR, False discovery rate; IQR, Interquartile range

Table S12. Comparison of metabolic changes over time with organ dysfunction in acute pancreatitis. The changes were calculated as the difference in concentration between two time points divided by the number of days in between. Significantly dysregulated lipids and other significantly altered metabolites are represented in bold.

Feature	No organ dysfunction, median [IQR]	Organ dysfunction, median [IQR]	p-value	FDR
Formate	0.002 [0 0.004]	0.003 [-0.002 0.004]	0.978	0.997
Unknown signal at 8.12 ppm	0.002 [-0.001 0.005]	0.001 [-0.001 0.006]	0.637	0.792
Unknown signal at 8.07 ppm	0 [0 0]	0.001 [0 0.003]	0.113	0.339
Phenylalanine	-0.005 [-0.014 0.013]	0.008 [0.003 0.014]	0.212	0.491
Tyrosine	0.001 [-0.003 0.005]	0 [-0.003 0.004]	0.718	0.852
Unknown signal at 7.14 ppm	0 [-0.001 0.021]	0 [-0.005 0.013]	0.597	0.780
Histidine	-0.005 [-0.008 0.003]	0.004 [-0.003 0.006]	0.063	0.248
Glucose	-0.053 [-0.291 0.266]	0.068 [-0.33 0.502]	0.524	0.722
Mannose	-0.001 [-0.014 0.005]	0.002 [-0.007 0.012]	0.332	0.604
Unknown signal at 5.15 ppm	0 [0 0]	0 [0 0]	0.462	0.722
Unknown signal at 5.09 ppm	0 [-0.001 0.001]	0.003 [0 0.008]	0.283	0.535
Unknown signal at 5.01 ppm	0 [0 0]	0 [0 0]	0.492	0.722
Ascorbate	0 [0 0.004]	0 [-0.005 0]	0.192	0.466
Threonine	0.004 [-0.006 0.029]	0.003 [-0.003 0.014]	0.978	0.997
Lactate	0.019 [-0.197 0.365]	0.068 [-0.15 0.158]	1.000	1.000
Creatinine	-0.003 [-0.011 0.005]	0 [-0.007 0.007]	0.978	0.997
Creatine	-0.004 [-0.017 0]	-0.003 [-0.021 0]	0.934	0.997
Glycine	0.013 [-0.003 0.028]	0.035 [0.026 0.045]	0.081	0.257
Methanol	0.002 [-0.005 0.029]	0.001 [-0.001 0.002]	0.524	0.722
Unknown signal at 2.55 ppm	0 [0 0]	0.004 [0 0.058]	0.027	0.140
Citrate	-0.007 [-0.016 0.002]	0.001 [-0.015 0.008]	0.421	0.693
Glutamine	-0.003 [-0.043 0.043]	0.019 [0.012 0.028]	0.390	0.663
Pyruvate	-0.01 [-0.022 0.002]	0.006 [0 0.022]	0.071	0.257
Glutamate	0.025 [0 0.05]	0.029 [0.018 0.063]	0.524	0.722
**Acetoacetate**	**0.001 [-0.022 0.008]**	**0.094 [0.043 0.231]**	**0.007**	**0.121**
Acetate	0 [-0.003 0.007]	0.003 [0 0.005]	0.978	0.997
Alanine	0.015 [-0.032 0.05]	0.002 [-0.014 0.027]	0.637	0.792
Unknown signal at 1.45 ppm	0 [-0.005 0.006]	0.002 [-0.005 0.014]	0.524	0.722
Unknown signal at 1.43 ppm	0 [0 0]	0 [0 0]	0.175	0.447
**3-Hydroxybutyrate**	**0.001 [-0.046 0.005]**	**0.275 [0.033 0.599]**	**0.011**	**0.134**
Ethanol	0 [0 0.009]	0 [0 0]	0.264	0.518
Unknown signal at 1.16 ppm	0 [-0.082 0.023]	0 [-0.055 0]	0.652	0.792
Unknown signal at 1.14 ppm	0 [-0.011 0.017]	0 [-0.003 0.004]	0.889	0.997
Unknown signal at 1.11 ppm	-0.01 [-0.014 -0.002]	-0.002 [-0.004 0.003]	0.142	0.380
Unknown signal at 1.06 ppm	-0.003 [-0.006 0.003]	0.003 [-0.006 0.008]	0.255	0.518
Valine	-0.003 [-0.02 0.014]	0.019 [-0.003 0.045]	0.127	0.360
Isoleucine	0 [-0.007 0.01]	0.011 [0.002 0.014]	0.081	0.257
**Leucine**	**-0.002 [-0.008 0.007]**	**0.009 [0.002 0.02]**	**0.038**	**0.159**
2-Hydroxybutyrate	-0.007 [-0.015 0]	-0.003 [-0.016 0]	0.560	0.751
Protein NH	1.147 [-6.205 6.871]	-1.031 [-5.977 7.473]	0.890	0.997
**Unsaturated lipid -CH=CH-**	**-0.247 [-0.625 0.088]**	**0.786 [0.505 1.611]**	**0.018**	**0.134**
**Lipid alpha-CH_2**	**-0.271 [-0.361 0.03]**	**0.418 [0.045 0.603]**	**0.002**	**0.106**
**Cholesterol**	**-0.004 [-0.061 0.039]**	**0.052 [0.03 0.156]**	**0.021**	**0.136**
**Lipid =CH-CH_2-CH=**	**-0.113 [-0.215 0.051]**	**0.351 [0.151 0.636]**	**0.018**	**0.134**
**Glycorol phospholipid**	**-0.11 [-0.279 0]**	**0.033 [-0.002 0.138]**	**0.025**	**0.140**
Phospholipid	-0.003 [-0.313 0.135]	0.063 [-0.008 0.206]	0.255	0.518
**Lipid beta-CH_2**	**-0.149 [-0.514 0.041]**	**0.42 [0.112 1.023]**	**0.014**	**0.134**
**Lipid CH_2**	**-2.623 [-5.925 0.397]**	**5.321 [0.886 9.455]**	**0.007**	**0.121**
**Lipid CH_3**	**0.117 [-1.375 0.627]**	**1.104 [0.486 2.753]**	**0.038**	**0.159**
GlycB	0.012 [0 0.037]	0.042 [0.028 0.056]	0.233	0.517
GlycA	0.019 [-0.034 0.18]	0.132 [0.037 0.279]	0.360	0.633

Abbreviations: FDR, False discovery rate; IQR, Interquartile range
